# Sulphonamide inhibition studies of the β-carbonic anhydrase GsaCAβ present in the salmon platyhelminth parasite *Gyrodactylus salaris*

**DOI:** 10.1080/14756366.2023.2167988

**Published:** 2023-01-17

**Authors:** Ashok Aspatwar, Alessandro Bonardi, Heidi Aisala, Ksenia Zueva, Craig R Primmer, Jaakko Lumme, Seppo Parkkila, Claudiu T. Supuran

**Affiliations:** a Faculty of Medicine and Health Technology, Tampere University, Tampere, Finland; b Department of Neuroscience, Psychology, Drug Research and Child’s Health, Section of Pharmaceutical and Nutraceutical Sciences, University of Florence, Sesto Fiorentino, Italy; c Ecology and Genetics, University of Oulu, Oulu, Finland; d Department of Biology, University of Turku, Turku, Finland; e Institute of Biotechnology, University of Helsinki, Helsinki, Finland; f Organismal and Evolutionary Biology Research Programme, University of Helsinki, Helsinki, Finland; g Fimlab Ltd, Tampere University Hospital, Tampere, Finland

**Keywords:** Carbonic anhydrase, *Gyrodactylus salaris*, kinetics, sulphonamide inhibitors, sulfamate

## Abstract

A β-class carbonic anhydrase (CA, EC 4.2.1.1) present in the genome of the Monogenean platyhelminth *Gyrodactylus salaris,* a fish parasite, GsaCAβ, has been investigated for its inhibitory effects with a panel of sulphonamides and sulfamates, some of which in clinical use. Several effective GsaCAβ inhibitors were identified, belonging to simple heterocyclic sulphonamides, the deacetylated precursors of acetazolamide and methazolamide (*K*_I_sof 81.9–139.7 nM). Many other simple benezene sulphonamides and clinically used agents, such as acetazolamide, methazolamide, ethoxzolamide, dorzolamide, benzolamide, sulthiame and hydrochlorothiazide showed inhibition constants <1 µM. The least effective GsaCAβ inhibitors were 4,6-disubstituted-1,3-benzene disulfonamides, with *K*_I_s in the range of 16.9–24.8 µM. Although no potent GsaCAβ-selective inhibitors were detected so far, this preliminary investigation may be helpful for better understanding the inhibition profile of this parasite enzyme and for the potential development of more effective and eventually parasite-selective inhibitors.

## Introduction

We have recently reported the cloning and characterisation of a β-class carbonic anhydrase (CA, EC 4.2.1.1) encoded in the genome of *Gyrodactylus salaris*, GsaCAβ^1^, a platyhelminth (flatworm) parasite attacking various fish species^2^^,^^3^. The Atlantic salmon (*Salmo salar*) is particularly sensitive to this parasite, which produced catastrophic losses in fish farms in Scandinavian countries and elsewhere, starting with the 1970s^3–5^. By releasing proteolytic enzymes, the parasite attaches on the fish gills, fins or skin inducing the formation of wounds, which favour the emergence of infections, with debilitation and eventual death of the infected animals^5^^,^^6^. There are no effective drugs for the treatment of this parasitic disease, although a variety of inorganic salts, synthetic compounds/drugs (e.g., praziquantel, levamisole, mebendazole and toltrazuril) and other approaches (manual removal of the worms) have been investigated, with rather unsuccessful results^7^. Furthermore, many of these compounds/drugs induce serious host toxicity, raising thus significant human health concerns if such fish is to be consumed^7^. Thus, as for other platyhelminth parasites producing infection in vertebrates including humans, such as *Schistosoma haematobium*^8^ or *Schistosoma mansoni*^9–11^ there is a stringent need of alternative drug targets and efficient compounds to treat these infections.

**Figure 1.**
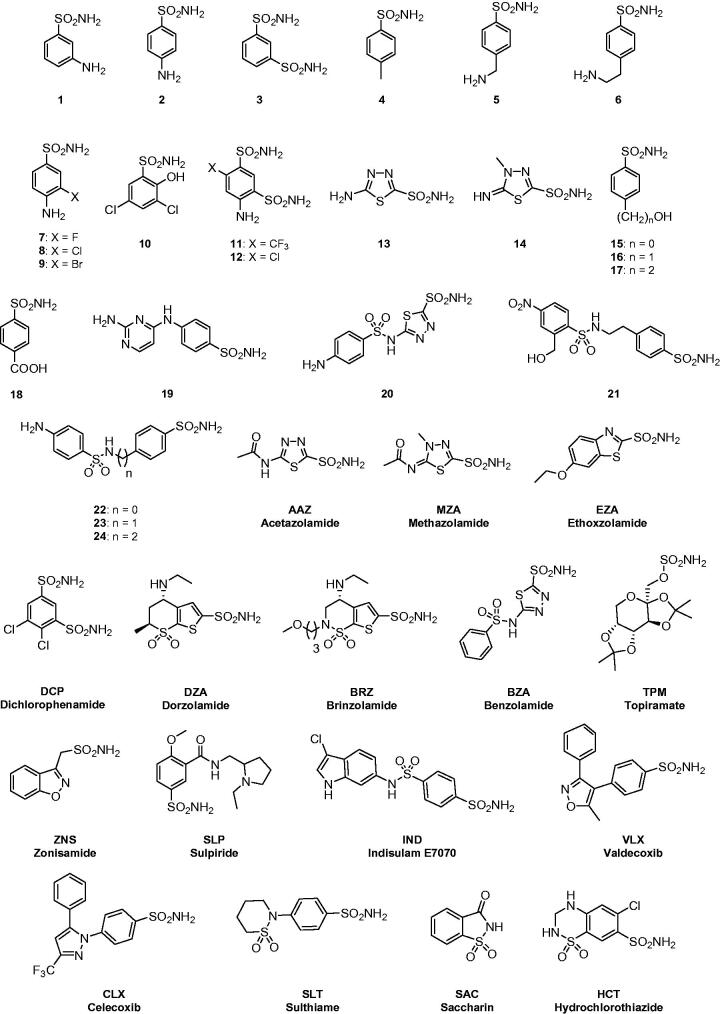
Sulphonamides/sulfamates **1–24** and **AAZ-HCT** investigated as inhibitors in the present study.

CAs are well known drug targets for the management of human diseases^12–15^, with their inhibitors acting as diuretics^16^, antiepileptics^17^, antiglaucoma^18^, antiobesity^19^ and antitumor agents^20^. In the last decade, CAs from pathogens started to be considered as possible targets for the development of antiinfectives, for the management of diseases provoked by bacteria^21^, fungi^22^, protozoa^23^ and worms^10^^,^^11^^,^^24^. In the previous work^1^ we have shown that GsaCAβ has a significant catalytic activity for the physiologic, CO_2_ hydration reaction, with a *k*_cat_ of 1.1 × 10^5^ s^−1^ and a *k*_cat_/*K_m_* of 7.58 × 10^6^ M^−1^ × s^−1^. Furthermore, inorganic anions, a well-known class of CA inhibitors (CAIs)^14^^,^^15^ inhibit the enzyme in the millimolar range, as for other α- and β-CAs investigated for their interaction with such modulators of activity^14^. Among the investigated such inhibitors, sulfamide (K_I_ of 81 µM) and sulphamic acid (*K_I_* of 6.2 µM) showed the most efficient inhibitory action^1^. Both of them incorporate the SO_2_NH_2_ moiety found in the most investigated class of CAIs, the aromatic/heterocyclic sulphonamides and their isosteres (sulfamates, sulfamides)^14^^,^^15^. Thus in this work we report GsaCAβ inhibition studies with a panel of such compounds, many of which are clinically used drugs (Figure 1).

## Materials and methods

### Chemistry

Compounds **1–24** and **AAZ-HCT** were commercially available, highest purity reagents from Sigma-Aldrich (Milan, Italy) or were synthesised as previously reported^25^.

### Production of β-CA recombinant protein

Protein production was carried out according to the previously reported protocol^1^.

### Ca activity and inhibition measurements

An Applied Photophysics stopped-flow instrument has been used for assaying the CA catalysed CO_2_ hydration activity^26^. Phenol red at a concentration of 0.2 mM was used as pH indicator, working at the absorbance maximum of 557 nm, with 10 mM TRIS (pH 8.3) as buffer, and in the presence of 10 mM NaClO_4_ for maintaining constant the ionic strength, following the initial rates of the CA-catalysed CO_2_ hydration reaction for a period of 10–100 s. The CO_2_ concentrations ranged from 1.7 to 17 mM for the determination of the kinetic parameters and inhibition constants. For each inhibitor, at least six traces of the initial 5–10% of the reaction have been used for determining the initial velocity. The uncatalyzed rates were determined in the same manner and subtracted from the total observed rates. Stock solutions of inhibitors (10–20 mM) were prepared in distilled-deionized water and dilutions up to 0.01 µM were done thereafter with the assay buffer. Inhibitor and enzyme solutions were preincubated together for 15 min at room temperature prior to assay, in order to allow for the formation of the enzyme-inhibitor complex. The inhibition constants were obtained by non-linear least-squares methods using PRISM 3 and the Cheng-Prusoff equation, whereas the kinetic parameters for the uninhibited enzymes from Lineweaver-Burk plots, as reported earlier^27^^,^^28^, and represent the mean from at least three different determinations. GsaCAβ concentration in the assay system was of 11.9 nM.

## Results and discussion

GsaCAβ shows catalytic properties for the physiologic reaction similar to those of the slow human isoform hCA I, being however slightly less effective as a catalyst compared to hCA I (Table 1). On the other hand, it should be stressed that many CAs are among the most effective catalysts known in nature^14^^,^^15^, and even this level of activity is in fact quite significant.

**Table 1.** Kinetic parameters for the CO_2_ hydration reaction catalysed by α- and β-class CA enzymes: the human cytosolic isozymes hCA I and II (α-class) at 20 °C and pH 7.5 in 10 mM HEPES buffer, and GsaCAβ (measured at 20 °C, pH 8.3 in 20 mM TRIS buffer and 10 mM NaClO_4_) are shown. Inhibition data with the clinically used sulphonamide acetazolamide are also presented.

Isozyme	Activity level	*k*_cat_	*k*_cat_/*K_m_*	*K_I_* (acetazolamide)
		(s^−1^)	(M^−1^ × s^−1^)	(nM)
hCA I^a^	Moderate	2.0 × 10^5^	5.0 × 10^7^	250
hCA II^a^	Very high	1.4 × 10^6^	1.5 × 10^8^	12
GsaCAβ^b^	Low-moderate	1.1 × 10^5^	7.58 × 10^6^	460.5

^a^From ref. [12,15]; ^b^From ref. [1].

We have investigated the inhibition profile of GsaCAβ with a panel of sulphonamides and sulfamates (Figure 1) known to effectively inhibit many classes of CAs, with some of these derivatives being clinically used drugs for decades, in the treatment of a multitude of diseases, as shown in the introduction. The names of the relevant drugs are reported in Figure 1, and as mentioned above, they are used as diuretics, antiglaucoma drugs, antiepileptics or for the management of other disorders connected with CA activity disbalances^14^^,^^15^. The GsaCAβ inhibition data with these compounds, as well as those for hCA I and II (for comparison reasons), are shown in Table 2.

**Table 2.** Inhibition of β-CA from *G. salaris* and human isoforms hCA I and hCA II with sulphonamides **1–24** and the clinically used drugs **AAZ-HCT**, by a stopped-flow assay^26^.

	*K_I_* (nM)^a^		
Inhibitor	hCA I	hCA II	GsaCAβ
1	28,000	300	522.8
2	25,000	240	589.2
3	79	8	388.8
4	78,500	320	3115
5	25,000	170	2144
6	21,000	160	7790
7	8300	60	854.4
8	9800	110	7266
9	6500	40	8879
10	7300	54	9103
11	5800	63	16900
12	8400	75	24820
13	8600	60	81.9
14	9300	19	139.7
15	5500	80	419.8
16	9500	94	616.1
17	21,000	125	917.4
18	164	46	687.6
19	109	33	489.1
20	6	2	631.8
21	69	11	5839
22	164	46	765.9
23	109	33	653.2
24	95	30	382.2
AAZ	250	12	460.5
MZA	50	14	721.7
EZA	25	8	545.9
DCP	1200	38	3261
DZA	50,000	9	399.1
BRZ	45,000	3	5063
BZA	15	9	716.3
TPM	250	10	8558
ZNS	56	35	8576
SLP	1200	40	7288
IND	31	15	7423
VLX	54,000	43	3892
CLX	50,000	21	4621
SLT	374	9	877.1
SAC	18,540	5959	1635
HCT	328	290	776.8

^a^Mean from three different assays. Errors (data not shown) were in the range of ± 10% of the reported data.

As seen from Table 2, where the inhibition data of the human α-class isoforms hCA I and II were also included for comparison, all investigated sulphonamides/sulfamates inhibited GsaCAβ, with inhibition constants raging between 81.9 nM and 24.8 µM. The following structure-activity relationship (SAR) should be noted regarding the inhibition data of Table 2:

- The most effective GsaCAβ inhibitors were compounds **13** and **14**, the deacetylated precursors of acetazolamide and methazolamide, which showed *K_I_* values of 81.9–139.7 nM, which is 5.1–5.6 times a better inhibitory activity compared to the clinically used derivatives **AAZ** and **MZA** (Table 2). As seen in Table 2, these precursors are less effective as hCA I and II inhibitors compared to the acetylated derivatives used as drugs.
- A rather large number of derivatives, such as **1–3, 7, 15–20, 2–24, AAZ, MZA, EZA, DZA**, **BZA**, **SLT** and **HCT**, showed less effective inhibition, but anyhow with K_I_s <1000 nM. The SAR is rather difficult to rationalise in this case as these compounds belong to very heterogeneous classes of sulphonamides, both aromatic (benzene sulphonamides) and heterocyclic derivatives. However, it seems that rather simple and elongated scaffolds lead to effective inhibition whereas the inclusion of bulkier substituents (e.g. in **21** compared to **22–24**, or **BRZ** compared to **DZA**) is detrimental for the inhibitory activity.
- Compounds showing low micromolar inhibition of GsaCAβ were **4–6, 8–10, 21, DCP, BRZ, TPM, ZNS, SLP; IND, VLX, CLX** and **SAC.** These compounds had *K*_I_s in the range of 1.63–9.1 µM. As above, they belong to a large number of diverse chemotypes in order to draw a rationalisation of their SAR. Saccharin, also being a medium potency inhibitor, is among the most selective ones for inhibiting GsaCAβ over the human isoforms (Table 2).
- 4,6-disubstituted-1,3-benzene disulfonamides **11** and **12** were the least effective GsaCAβ inhibitors, with *K*_I_s in the range of 16.9–24.8 µM (Table 2).
- The inhibition profile of GsaCAβ and hCA I/II are very different, obviously due to the fact that they belong to diverse genetic CA families. Unfortunately, no GsaCAβ-selective inhibitors (over the hCAs investigated here) were detected so far.

## Conclusions

The Monogenean platyhelminth *Gyrodactylus salaris,* a fish parasite of salmon and other economically relevant aquaculture fish species, encodes for a β-class CA, GsaCAβ, which has been investigated here for its inhibition profile with sulphonamides/sulfamates, as a possible antiparasitic drug target. We identified several effective GsaCAβ inhibitors, belonging to simple heterocyclic sulphonamide derivatives, the deacetylated precursors of acetazolamide and methazolamide, which showed *K_I_* values of 81.9 − 139.7 nM. Many other simple benezenesulfonamides and clinically used agents, such as acetazolamide, methazolamide, ethoxzolamide, dorzolamide, benzolamide, sulthiame and hydrochlorothiazide showed inhibition constants <1 µM. The least effective GsaCAβ inhibitors were 4,6-disubstituted-1,3-benzene disulfonamides, with *K_I_*s in the range of 16.9 − 24.8 µM. Although no GsaCAβ-selective inhibitors were detected so far, this preliminary investigation may be helpful for better understanding the SAR for inhibition of this parasite enzyme and for the potential development of more effective and eventually parasite-selective inhibitors.
